# Associations between unfulfilled contraceptive preferences due to cost and low-income patients’ access to and experiences of contraceptive care in the United States, 2015–2019^☆^^☆☆^

**DOI:** 10.1016/j.conx.2022.100076

**Published:** 2022-05-06

**Authors:** Megan L. Kavanaugh, Emma Pliskin, Rubina Hussain

**Affiliations:** Guttmacher Institute, New York, NY, United States

**Keywords:** Contraceptive use, Contraceptive preferences, National Survey of Family Growth, United States, Person-centered contraceptive counseling, Cost

## Abstract

**Objective:**

To identify prevalence of unfulfilled contraceptive preferences due to cost among low-income United States female contraceptive method users and nonusers, and associations between access to, and experience with, contraceptive care and this outcome.

**Methods:**

We drew on data from the 2015–2019 National Surveys of Family Growth to conduct simple and multivariable logistic regression analyses on unfulfilled contraceptive preferences due to cost among nationally representative samples of low-income women ages 15 to 49 who were current contraceptive users (*N* = 3178) and nonusers (*N* = 1073).

**Results:**

Overall, 23% of female contraceptive users reported they would use a different method, and 39% of nonusers reported they would start using a method, if cost were not an issue. Controlling for user characteristics, low-income contraceptive users who received recent publicly supported contraceptive care reported significantly higher levels of unfulfilled contraceptive preferences due to cost than those without any access to SRH care (aOR = 1.6, CI 1.0–2.5), while having private (aOR = 0.6, CI 0.4–0.9) or public (aOR = 0.7, CI 0.5–1.0) health insurance was associated with significantly lower levels of this outcome. Nonusers of contraception who had recently received publicly supported contraceptive care also reported marginally higher levels of this outcome (aOR = 2.2, CI 1.0–5.1). Contraceptive users who received recent person-centered contraceptive counseling had marginally lower odds of unfulfilled contraceptive preferences due to cost (aOR = 0.6, CI 0.4–1.0).

**Conclusions:**

Cost is a barrier to using preferred contraception for both contraceptive users and nonusers; health insurance coverage and person-centered contraceptive counseling may help contraceptive users to overcome cost barriers and realize their contraceptive preferences.

**Implications:**

Factors related to contraceptive access at the systems level—specifically the subsidization and experience of contraceptive care—impact whether cost serves as a barrier to individuals’ contraceptive preferences. Delivery of patient-centered care and shoring up health insurance coverage for all can help to mitigate cost barriers and enable individuals to realize their contraceptive preferences.

## 1 Introduction

Cost plays a key role in people's access to contraception. As of 2015–2017, 1 in 5 women in the United States would prefer to use (another) contraceptive method if cost were not a consideration [1]. Cost, including affordability of methods and contraceptive-related health care, factors prominently in which methods individuals use [2] and why users may not be using their preferred method [3]. Financial resources—income and health insurance coverage—also play a role in individuals’ ability to realize their contraceptive preferences, with greater mismatches between preferred and used contraceptive methods among women with lower socioeconomic statuses than among those with higher incomes [4,5]. In a 2020 national study, 25% of women who were not using their preferred method of contraception indicated that it was because they could not afford it [6]. Among women at risk of unintended pregnancy in the United States, nonusers of contraception are more likely to have lower incomes and be uninsured compared to users [7].

Beyond cost and affordability, one's source and experience of contraceptive care can also influence contraceptive choices. Individuals who seek care at Title X–funded health care sites, for instance, have access to a broader range of contraceptive methods than those visiting non-Title X–funded sites [8]. Those getting care at sites that receive any public funding for contraceptive service delivery, where contraceptive care is more commonly low or no cost, have higher rates of overall contraceptive use as well as use of more effective methods compared to those getting care at sites that don't receive this public funding [8]. Finally, interactions with a health care provider during the contraceptive care visit, including one's perceptions of the patient-centeredness of the interaction [9], can impact becoming a contraceptive user or nonuser [10], which methods are used [11], and whether users are employing their preferred method [3]. Providers can play a role in facilitating dialogue during contraceptive counseling sessions about anticipated cost barriers to preferred contraception and troubleshooting how to navigate them ahead of time[12].

Given the evidence to date, our analysis documents the overall national prevalence of, and characteristics associated with, unfulfilled contraceptive preferences due to cost in the United States among low-income method users and nonusers for 2015–2019. We conceptualize unfulfilled contraceptive preferences as a desire to use a different method of contraception among contraceptive users, or to use any method of contraception among nonusers, if cost were not an issue. In addition, given the roles that both access to, and experience with, contraceptive care may play with regards to cost-related barriers to contraceptive access, and thus ability to fulfill preferences, we examine associations between these key indicators and individuals’ unfulfilled contraceptive preferences due to cost.

## 2 Methods

Data for this cross-sectional, descriptive study come from the female respondent files of the 2015–2017 and 2017–2019 National Survey of Family Growth (NSFG), which provides the most comprehensive nationally representative information available on contraceptive use in the United States.^1^ The NSFG conducts in-home, face-to-face interviews of civilian, noninstitutionalized women aged 15 to 49 and uses a multistage probability sampling design that oversamples Black and Hispanic groups and adolescents aged 15 to 19. More detailed information on survey methodology, sample design, response rates, fieldwork procedures, and variance estimation is published elsewhere^2^, and the data are available for download on the NSFG website.^3^ Given the de-identified nature of these public use data, our organization's institutional review board (Department of Health and Human Services identifier IRB00002197) determined that all analyses drawing on NSFG data are exempt from institutional review board approval.

Our primary outcome of interest in this study is unfulfilled contraceptive preference due to cost. This outcome drew on data from contraceptive users responding to the question “*If you did not have to worry about cost and could use any type of contraceptive method available, would you want to use a different method?*” and nonusers of contraception responding to “*If you did not have to worry about cost and could use any type of contraceptive method available, would you want to use a method?*” We determined contraceptive use status according to whether respondents reported using any contraceptive method during the month of survey interview (current users) or not (nonusers of contraception). We maintain distinctions between contraceptive users and nonusers of contraception in examining our outcome of interest due to key differences in demographic and sexual and reproductive health (SRH) characteristics between these 2 groups [7], which may have implications for our analysis.

We focus on 2 key independent variables as representing access to SRH care and influencing our outcome of interest: health care insurance coverage (none including only a single-service plan or only Indian Health Service coverage; private or Medi-Gap; or public including Medicaid, Medicare, military healthcare, Children's Health Insurance Program, or another government or state-sponsored health plan) and source of SRH-related care in the prior year. To represent whether a respondent had received SRH^4^-related care in the last year and, if so, the source of that care, we created a 4-category variable that classified respondents as having received: (1) no SRH care, (2) SRH care that wasn't contraceptive care, (3) contraceptive-specific SRH care at a private site, and (4) contraceptive-specific SRH care at a public site. Respondents who had received contraceptive-specific SRH care in the last year at a public clinic (via Title X or alternative public funding) were considered to have received publicly supported contraceptive care and those receiving any of those services at a private doctor or HMO were considered to have received private contraceptive-specific SRH care, prioritized in that order for respondents who reported visits for both of those types of care in the past year^5^ [13].

For our third key independent variable representing contraceptive care experiences, we relied on 4 newly introduced items to the 2017–2019 NSFG assessing respondents’ experiences with contraceptive care, together considered to represent the Person-Centered Contraceptive Counseling (PCCC) measure [9]. The PCCC items asked respondents who received a method of birth control or contraceptive counseling in the past 12 months to rate on a 5-point Likert scale whether their provider respected them as a person, let them say what mattered most about their birth control, took their preferences about birth control seriously, and gave them enough information to make the best decision about their birth control. Based on published guidance from the team who developed the PCCC, we combined these 4 items to create a dichotomous variable that considered those who rated their provider as “excellent” on all 4 characteristics to have received person-centered contraceptive counseling, while those who rated their provider as anything less than “excellent” on any were considered to have not [14,15].

In addition to the key independent variables of interest described above, we also consider several demographic and SRH characteristics that may be associated with contraceptive method use and preferences: age, race and ethnicity, nativity, relationship status, education, parity, and sexual identity. Among current contraceptive users, we also categorized the type of method used during the month of interview (prioritizing the single, most effective method used when multiple methods were reported): long-acting reversible contraceptives (LARCs—IUDs and implants); short-acting reversible contraceptives (SARCs—pills, the patch, the shot, and the ring); and condoms and all other methods.

To examine characteristics associated with unfulfilled contraceptive preferences due to cost, we pooled data from the 2015–2017 and 2017–2019 female respondent files of the NSFG and weighted the data to reflect the United States reproductive-aged female civilian population for each of the 2-year time spans—September 2015 to September 2017 and September 2017 to September 2019. Our primary outcome focuses on cost as a barrier to realizing contraceptive preferences; as such we limit our analysis to low-income female respondents who reported income levels of <300% of the federal poverty level (FPL). Our analytic sample included 4251 low-income females aged 15 to 49 who reported their current contraceptive use status and had responded to the corresponding unfulfilled contraceptive preferences item. We excluded individuals who reported using permanent contraception (own or their partner's) or who indicated that they or their partner were sterile due to noncontraceptive reasons and, among nonusers of contraception, those who indicated that they did not have a male sexual partner in the past 12 months or who were actively trying to become pregnant as a reason for not using contraception.

We first examined distributions of sociodemographic and SRH characteristics among all women aged 15 to 49 and then broken down by current contraceptive users and nonusers of contraception, using Pearson's *X*^2^ to test differences among the 2 subpopulations. Within each of these groups, we examined the percentages who reported unfulfilled contraceptive preferences due to cost by sociodemographic and SRH characteristics and then used simple logistic regression to examine bivariate relationships between characteristics and our outcome of interest.

Next, we used multivariable logistic regression to estimate adjusted odds ratios for the relationship between respondent characteristics and unfulfilled contraceptive preferences due to cost, separately for contraceptive users and nonusers. For each of these groups, we ran 2 staged models to assess the relationships between individuals’ sociodemographic and SRH characteristics and their unfulfilled contraceptive preferences due to cost, with specific attention to the role played by individuals’ access to, and experiences with, contraceptive care. The first set of models included sociodemographic and SRH characteristics with theoretical and evidence-based relevance to contraceptive use, including the 2 key variables representing access to SRH care (health care insurance coverage and source of SRH care). The second set of models included the same variables in the first model, with the addition of the composite PCCC measure to understand the additional relationship between patients’ experiences with contraceptive care and unfulfilled contraceptive preferences due to cost, controlling for respondent demographic and SRH characteristics. These second models narrowed the analytic sample to only those who had reported getting contraceptive care in the prior year and drew on data only from 2017 to 2019, as the PCCC items were only asked of those who got care and were not included in previous NSFGs. We highlight adjusted odds ratios (aORs) of associations and their 95% confidence intervals (CIs) in models significant at, or close to, the *p* < 0.05 level.

All analyses were conducted using the “svy” command prefix within Stata 16.1 to account for the NSFG's use of a multistage probability sample.

## 3 Results

### 3.1 Characteristics of the sample

Of the 4251 eligible low-income women aged 15 to 49 in the 2015–2019 study population, 75% were currently using contraception and 25% were nonusers (Table 1). Characteristics of both contraceptive users and nonusers of contraception roughly aligned with characteristics of the full sample, with regards to experiences with patient-centered contraceptive care, nativity status, relationship status, educational attainment, and sexual identity. Contraceptive users and nonusers of contraception demonstrated greater variation in their source of SRH care, health insurance coverage, age, income and parity.

**Table 1:** Distributions of selected access and experiences of sexual and reproductive health and sociodemographic characteristics among analytic sample, overall and by contraceptive use status, National Survey of Family Growth 2015–2019

	Full sample		Women currently using contraception		Women not currently using contraception a		
	N	%	N	%	N	%	*p*-value
Overall^b^	4251	100%	3178	75%	1073	25%	
							
Access and experiences of SRH							
Current method used^c^							*p* < 0.001
No method	1073	25%	0	0%	1073	100%	
LARC methods	767	18%	767	24%	0	0%	
SARC methods	1169	27%	1169	36%	0	0%	
Condom and other methods	1242	30%	1242	39%	0	0%	
Source of SRH care^d^							*p* < 0.001
No SRH care	756	19%	535	18%	221	23%	
SRH but no contraceptive care	1135	25%	635	19%	500	44%	
Private contraceptive-specific SRH care	1709	43%	1469	49%	240	23%	
Public contraceptive-specific SRH care	651	13%	539	14%	112	10%	
Current insurance coverage							*p* < 0.001
None	706	16%	524	15%	182	17%	
Private	1824	50%	1444	53%	380	42%	
Public	1721	34%	1210	32%	511	41%	
Composite patient-centered contraceptive counseling experience^e^							0.59
No	643	53%	543	53%	100	56%	
Yes	553	47%	487	47%	66	44%	
							
Demographic characteristics							
Age							*p* < 0.001
15–19 y	589	13%	477	15%	112	8%	
20–29 y	1788	41%	1361	43%	427	38%	
30–39 y	1350	29%	998	29%	352	31%	
40–49 y	524	16%	342	14%	182	23%	
Race/ethnicity							0.07
White, non-Hispanic	1539	48%	1213	49%	326	45%	
Black, non-Hispanic	1159	19%	825	17%	334	22%	
Other/multiple, non-Hispanic	350	8%	260	8%	90	8%	
Hispanic	1203	25%	880	25%	323	25%	
Federal poverty level							0.02
0%–99%	1665	36%	1189	34%	476	40%	
100%–199%	1526	37%	1159	37%	367	38%	
200%–299%	1060	27%	830	29%	230	22%	
Nativity status							0.84
US born	3524	83%	2643	83%	881	83%	
Foreign born	725	17%	534	17%	191	17%	
Relationship status							0.07
Married	1083	31%	774	29%	309	35%	
Cohabitating	670	18%	532	19%	138	15%	
Not married or cohabitating	2498	51%	1872	51%	626	50%	
Educational attainment							0.24
Not a high school graduate	813	17%	596	17%	217	17%	
High school graduate/GED	1395	30%	1023	29%	372	34%	
Some college	1354	35%	1036	36%	318	32%	
College graduate	689	18%	523	19%	166	17%	
Parity							0.01
0	1619	40%	1267	42%	352	35%	
1 or more	2630	60%	1910	58%	720	65%	
Sexual identity							0.34
Straight	3616	87%	2683	86%	933	88%	
Not straight	571	13%	449	14%	122	12%	

SRH, sexual and reproductive health; NSFG, National Survey of Family Growth; LARC, long-acting reversible methods; SARC, short-acting reversible methods; FPL, federal poverty level; IUD, intrauterine device; STI, sexually transmitted infection; PCCC, patient-centered contraceptive counseling.

Population includes all female respondents aged 15 to 49 at the time of interview who were under 300% of the FPL and who responded to the unfulfilled contraceptive preferences due to cost variables; population is weighted to reflect the female civilian population of the United States. Population excludes those who were sterile and whose partner was sterile for non-contraceptive purposes; those who used permanent methods such as tubal ligation, hysterectomy, or vasectomy as their most effective method; those who were not using any method of contraception and did not have a male sexual partner in the past 12 months; and those who were not using any method of contraception and were actively trying to become pregnant as a reason for not using contraception.

a *p*-values represent significant differences from Pearson's χ^2^ tests of association comparing the distribution of women using contraception and women not using contraception who are not trying to get pregnant by each SRH and demographic variable.

b Overall percentage represents the proportion of all women using and not using contraception

c LARC methods include IUDs and hormonal implants (Norplant, Implanon, or Nexplanon). SARC methods include pills, Depo-Provera and other injectables, the contraceptive patch (Ortho-Evra or Xulane), and the vaginal contraceptive ring. Other methods include noncondom coital methods such as withdrawal, the diaphragm, foam, jelly or cream, and emergency contraception; natural family planning methods such as periodic abstinence, cervical mucus tests, temperature rhythm, or calendar rhythm; and other nonspecified methods.

d Source of SRH care categorizes the clinic where the respondents received SRH care in the past 12 months. This includes gynecologic care, pregnancy care, STI care and contraceptive care. Contraceptive care includes contraceptive methods, contraceptive counseling, or a check-up related to contraceptive use.

e The composite PCCC measure combines all 4 patient-centered care items to create a dichotomous variable that considered those who rated their provider as “excellent” on all 4 characteristics to have received patient-centered contraceptive counseling, while those who rated their provider as anything less than “excellent” on any 1 of the 4 characteristics were considered to have not. This measure includes only respondents from survey years 2017–2019, as these were the only years this variable was asked in the NSFG.

### 3.2 Characteristics associated with unfulfilled contraceptive preferences due to cost

#### 3.2.1 Contraceptive users

Out of all low-income contraceptive users in 2015–2019, nearly 1 in 4 (23%) would prefer to use another method if cost was not a consideration (Table 2). At the bivariate level, we found that users of condoms or other methods as primary contraception have higher unfulfilled contraceptive preferences than LARC users. Recent access to contraceptive care via receiving this care at a private site vs receiving no SRH care or via private or public health insurance coverage was associated with lower odds of unfulfilled contraceptive preferences due to cost. Having recently received person-centered contraceptive counseling was also associated with reductions in this outcome. Identifying as Hispanic, falling into the lowest income level, and having been born outside the United States were all individually associated with higher levels of unfulfilled contraceptive preferences due to cost.

**Table 2:** Weighted percentages, unadjusted and adjusted odds ratios from simple and multivariable logistic regression analyses assessing associations between selected access and experiences of sexual and reproductive health and sociodemographic characteristics and unfulfilled contraceptive preferences due to cost by contraceptive use status among lower income women aged 15 to 49, National Survey of Family Growth 2015–2019

	Unfulfilled contraceptive preferences due to cost among contraceptive users				Unfulfilled contraceptive preferences due to cost among nonusers of contraception			
	Users, *N* = 3178		Model 1, *N* = 3130	Model 2^a^, *N* = 1020	Nonusers, *N* = 1073		Model 1, *N* = 1054	Model 2^a^, *N* = 166
	Weighted %	OR (95% CI)	aOR (95% CI)	aOR (95% CI)	Weighted %	OR (95% CI)	aOR (95% CI)	aOR (95% CI)
Overall	23%				39%			
								
Access and experiences of SRH								
Current method used^b^								
LARC methods	14%	1.00	1.00	1.00				
SARC methods	18%	1.28 (0.91, 1.78)	**1.46 (1.00, 2.13)**	1.05 (0.60, 1.82)				
Condom and other methods	33%	**2.91 (2.04, 4.13)**	**3.70 (2.51, 5.45)**	**3.66 (1.96, 6.83)**				
Source of SRH care^c^								
No SRH care	26%	1.00	1.00		33%	1.00	1.00	
SRH but no contraceptive care	24%	0.89 (0.58, 1.37)	0.81 (0.52, 1.27)		35%	1.08 (0.67, 1.77)	1.26 (0.69, 2.32)	
Private contraceptive-specific SRH care	19%	**0.65 (0.46, 0.91)**	1.08 (0.72, 1.62)	1.00	46%	**1.71 (0.97, 3.01)**	1.75 (0.92, 3.32)	1.00
Public contraceptive-specific SRH care	30%	1.20 (0.78, 1.83)	**1.58 (1.00, 2.50)**	**1.46 (0.99, 2.16)**	60%	**3.06 (1.49, 6.26)**	2.21 (0.97, 5.05)	0.54 (0.20, 1.41)
Current insurance coverage								
None	35%	1.00	1.00	1.00	45%	1.00	1.00	1.00
Private	19%	**0.43 (0.31, 0.60)**	**0.60 (0.41, 0.88)**	**0.37 (0.16, 0.85)**	34%	**0.62 (0.37, 1.04)**	0.66 (0.36, 1.23)	**5.22 (0.94, 29.13)**
Public	24%	**0.57 (0.41, 0.80)**	**0.69 (0.48, 0.99)**	0.53 (0.23, 1.19)	42%	0.88 (0.54, 1.41)	0.81 (0.48, 1.35)	1.68 (0.51, 5.59)
Composite patient-centered contraceptive counseling experience^d^								
No	24%	1.00		1.00	44%	1.00		1.00
Yes	17%	**0.64 (0.42, 0.98)**		**0.64 (0.40, 1.02)**	57%	1.73 (0.73, 4.10)		2.11 (0.77, 5.73)
								
Demographic characteristics								
Age								
15–19 y	20%	0.98 (0.59, 1.61)	1.37 (0.69, 2.69)	3.77 (0.88, 16.20)	57%	**2.88 (1.27, 6.54)**	**2.71 (1.09, 6.76)**	na
20–29 y	24%	1.21 (0.77, 1.91)	1.31 (0.79, 2.19)	3.19 (0.90, 11.36)	47%	**1.95 (1.09, 3.49)**	1.46 (0.82, 2.57)	1.68 (0.33, 8.64)
30–39 y	22%	1.10 (0.68, 1.80)	1.15 (0.70, 1.89)	2.58 (0.71, 9.35)	31%	0.97 (0.54, 1.72)	0.78 (0.42, 1.47)	1.26 (0.20, 7.84)
40–49 y	21%	1.00	1.00	1.00	31%	1.00	1.00	1.00
Race/ethnicity								
White, non-Hispanic	19%	1.00	1.00	1.00	30%	1.00	1.00	1.00
Black, non-Hispanic	22%	1.20 (0.86, 1.69)	0.97 (0.68, 1.37)	0.65 (0.37, 1.15)	40%	1.55 (0.94, 2.56)	1.26 (0.78, 2.04)	0.59 (0.13, 2.59)
Other/multiple, non-Hispanic	21%	1.16 (0.72, 1.86)	0.93 (0.57, 1.51)	1.53 (0.79, 2.98)	45%	1.86 (0.83, 4.13)	1.99 (0.92, 4.32)	4.24 (0.86, 20.80)
Hispanic	31%	**1.97 (1.37, 2.84)**	**1.52 (0.98, 2.35)**	1.61 (0.82, 3.19)	52%	**2.53 (1.53, 4.18)**	**2.77 (1.61, 4.77)**	2.31 (0.58, 9.18)
Federal poverty level								
0%–99%	25%	**1.39 (1.00, 1.92)**	1.18 (0.83, 1.66)	1.02 (0.48, 2.19)	41%	0.96 (0.56, 1.64)	0.56 (0.31, 1.03)	0.34 (0.10, 1.13)
100%–199%	24%	1.30 (0.94, 1.79)	1.12 (0.79, 1.58)	0.80 (0.42, 1.51)	36%	0.81 (0.47, 1.38)	0.65 (0.38, 1.12)	0.79 (0.24, 2.60)
200%–299%	19%	1.00	1.00	1.00	42%	1.00	1.00	1.00
Nativity status								
US born	21%	1.00	1.00	1.00	39%	1.00	1.00	1.00
Foreign born	32%	**1.79 (1.37, 2.34)**	1.41 (0.96, 2.08)	1.77 (0.87, 3.63)	39%	0.98 (0.60, 1.61)	0.72 (0.40, 1.29)	1.25 (0.38, 4.11)
Relationship status								
Married	20%	1.00	1.00	1.00	30%	1.00	1.00	1.00
Cohabitating	26%	1.40 (0.96, 2.05)	1.46 (0.97, 2.19)	0.68 (0.30, 1.53)	38%	1.43 (0.83, 2.49)	1.23 (0.68, 2.24)	1.82 (0.41, 8.03)
Not married or cohabitating	23%	1.19 (0.88, 1.60)	**1.61 (1.11, 2.34)**	2.00 (0.90, 4.44)	46%	**2.04 (1.36, 3.07)**	1.55 (0.92, 2.61)	**3.96 (1.34, 11.74)**
Educational attainment								
Not a high school graduate	25%	1.00	1.00	1.00	47%	1.00	1.00	1.00
High school graduate/GED	24%	0.93 (0.67, 1.29)	1.09 (0.78, 1.54)	0.88 (0.47, 1.66)	42%	0.81 (0.48, 1.36)	1.00 (0.58, 1.72)	1.00 (0.21, 4.65)
Some college	22%	0.83 (0.58, 1.20)	1.11 (0.74, 1.68)	1.43 (0.83, 2.48)	36%	0.63 (0.38, 1.02)	0.83 (0.47, 1.46)	2.13 (0.29, 15.82)
College graduate	20%	0.74 (0.47, 1.15)	1.14 (0.67, 1.95)	1.05 (0.42, 2.67)	33%	**0.54 (0.31, 0.95)**	0.91 (0.49, 1.69)	0.17 (0.01, 2.02)
Parity								
0	21%	1.00	1.00	1.00	38%	1.00	1.00	1.00
1 or more	24%	1.15 (0.91, 1.46)	1.28 (0.92, 1.77)	1.70 (0.89, 3.26)	40%	1.04 (0.71, 1.54)	**1.81 (1.11, 2.97)**	1.88 (0.43, 8.29)
Sexual identity								
Straight	22%	1.00	1.00	1.00	38%	1.00	1.00	1.00
Not straight	26%	1.22 (0.85, 1.75)	1.18 (0.82, 1.72)	0.83 (0.44, 1.59)	49%	1.52 (0.88, 2.63)	1.45 (0.77, 2.72)	0.50 (0.13, 1.93)

OR, odds ratio; CI, confidence interval; aOR, adjusted odds ratio; na, not available due to insufficient cell size; SRH, sexual and reproductive health; NSFG, National Survey of Family Growth; LARC, long-acting reversible methods; SARC, short-acting reversible methods; FPL, federal poverty level; IUD, intrauterine device; STI, sexually transmitted infection; PCCC, patient-centered contraceptive counseling.

Population includes all female respondents aged 15 to 49 at the time of interview who were under 300% of the FPL and who responded to the unfulfilled contraceptive preferences due to cost variables; population is weighted to reflect the female civilian population of the United States. Population excludes those who were sterile and whose partner was sterile for non-contraceptive purposes; those who used permanent methods such as tubal ligation, hysterectomy, or vasectomy as their most effective method; those who were not using any method of contraception and did not have a male sexual partner in the past 12 months; and those who were not using any method of contraception and were actively trying to become pregnant as a reason for not using contraception.

Bold font indicates ORs and aORs significant at or close to, the *p* < 0.05 level.

a Models 2 are limited to those respondents who received contraceptive care in past 12 months and only include respondents from the 2017–2019 NSFG data.

b LARC methods include IUDs and hormonal implants (Norplant, Implanon, or Nexplanon). SARC methods include pills, Depo-Provera and other injectables, the contraceptive patch (Ortho-Evra or Xulane), and the vaginal contraceptive ring. Other methods include noncondom coital methods such as withdrawal, the diaphragm, foam, jelly or cream, and emergency contraception; natural family planning methods such as periodic abstinence, cervical mucus tests, temperature rhythm, or calendar rhythm; and other non-specified methods.

c Source of SRH care categorizes the clinic where the respondents received certain kinds of care in the past 12 months. This includes gynecologic care, pregnancy care, STI care and contraceptive care. Contraceptive care includes contraceptive methods, contraceptive counseling, or a check-up related to contraceptive use.

d The composite PCCC measure combines all 4 patient-centered care items to create a dichotomous variable that considered those who rated their provider as “excellent” on all 4 characteristics to have received patient-centered contraceptive counseling, while those who rated their provider as anything less than “excellent” on any 1 of the 4 characteristics were considered to have not.

In Model 1, after controlling for user characteristics, users of condoms or other methods (aOR = 3.7, CI 2.5–5.5) and SARC method users (aOR = 1.5, CI 1.0–2.3) had higher levels of unfulfilled contraceptive preferences due to cost compared to LARC method users. Those who reported having received contraceptive care at a public facility also had higher odds of unfulfilled contraceptive preferences (aOR = 1.6, CI 1.0–2.5) compared to those who had not received any SRH care. Compared to those reporting no health insurance coverage, those with private (aOR = 0.6, CI 0.4–0.9) or public (aOR = 0.7, CI 0.5–1.0) health insurance coverage had lower levels of unfulfilled contraceptive preferences due to cost.

When narrowing the model to individuals who reported having received contraceptive care in the prior year in the 2017–2019 NSFG dataset, condom or other method users continued to have higher odds of unfulfilled contraceptive method preferences due to cost compared to LARC users (aOR = 3.7, CI 2.0–6.8), but SARC method users were no longer significantly different. Access to contraceptive care via receiving this care at a publicly support site remained marginally significantly associated with unfulfilled contraceptive preferences due to cost (aOR = 1.5, CI 1.0–2.2). Having private health insurance coverage remained significantly associated with reduced odds of unfulfilled contraceptive preferences due to cost (aOR = 0.4, CI 0.2–0.9), although publicly insured individuals were no longer significantly different on this outcome from non-insured ones. Among contraceptive users, having experienced excellent person-centered contraceptive counseling overall based on the composite PCCC measure was marginally associated with reduced odds of reporting unfulfilled contraceptive preferences due to cost after controlling for all other SRH and demographic characteristics (aOR = 0.6, CI 0.4–1.0). In this model focused on low-income contraceptive users who had received contraceptive care in the past year, no demographic characteristics remained significantly associated with the outcome of interest.

#### 3.2.2 Nonusers of contraception

Overall, 39% of all low-income nonusers of contraception would prefer to use a method if cost was not a factor. At the bivariate level, we found an opposite relationship between source of SRH care and having unfulfilled contraceptive preferences due to cost among nonusers of contraception as compared to users; having received private or public contraceptive care were each at least marginally significantly associated with increased odds of unfulfilled contraceptive preferences due to cost. As with users, nonusers with private health insurance coverage had marginally lowered unfulfilled contraceptive preferences due to cost than those with no coverage. Several demographic characteristics of nonusers of contraception, including young age between 15 and 29, identifying as Hispanic, and not being married or cohabiting, were associated with higher levels of the outcome as well. Having at least some college education was at least marginally associated with lower unfulfilled contraceptive preferences due to cost.

In Model 1, after controlling for other characteristics of nonusers of contraception, those who had received recent public contraceptive care continued to have marginally higher levels of unfulfilled contraceptive preferences due to cost (aOR = 2.2, CI 1.0–5.1). Young age, identifying as Hispanic and having had a child were all demographic characteristics of nonusers of contraception associated with higher levels of unfulfilled contraceptive preferences due to cost at the multivariable level. Being in the lowest income level among nonusers of contraception was marginally associated with reduced odds of this outcome (aOR = 0.6, CI 0.3–1.0)

When further narrowing Model 2 to only those lower income individuals who had received contraceptive care in the past year, having private health insurance coverage was marginally associated with higher levels of unfulfilled contraceptive preferences compared to those who did not have any insurance coverage (aOR = 5.2, CI 0.9–29.1). There was no significant association between contraceptive nonusers’ experiences of person-centered contraceptive counseling and their unfulfilled contraceptive preferences due to cost. Among lower income nonusers of contraception who had gotten contraceptive care in the past year, being neither married nor cohabitating with a partner was associated with significantly higher odds of unfulfilled contraceptive preferences due to cost than being married (aOR = 4.0, CI 1.3–11.7). Given the reduced N of this final model among nonusers of contraception (*N* = 166), these findings should be interpreted with caution.

## 4 Discussion

Among low-income reproductive-aged women in the United States in 2015–2019, almost one-quarter of contraceptive users and nearly 4 in 10 nonusers of contraception had unfulfilled contraceptive preferences due to cost. Our findings are in comparison to a recent national study, which identified 18% of all women who would prefer to use a different method than the current one, with one-quarter of these indicating that they weren't using their preferred method because they couldn't afford it [6]. Our findings highlight that low-income contraceptive users and nonusers experience cost-related barriers to realizing reproductive autonomy through choice of a preferred contraceptive method. For nonusers, especially, these cost barriers prevent them from not just using their preferred method of contraception, but any method at all.

Health insurance emerged as a key driver of lowered unfulfilled contraceptive preferences due to cost among both users and nonusers of contraception. National [16] and state-level [17] data indicate that individuals with health insurance have higher levels of contraceptive use than those with no coverage. Efforts aimed at reducing the cost burden on individuals for health care, especially via health insurance that covers a broad range of contraceptive methods such as that guaranteed under the ACA, contribute to individuals being able to realize reproductive autonomy with regards to choosing—and using—preferred methods of contraception. Having health insurance coverage may not be enough to shift nonusers who would like to be using a method into becoming a user, for reasons that this analysis is unable to detect.

On the other hand, both users and nonusers of contraception who had received publicly supported contraceptive care had higher levels of unfulfilled contraceptive preferences due to cost, highlighting the clear barrier that cost plays for those with the fewest resources to overcome it, even in settings that are set up to minimize these barriers for low-income individuals. Publicly supported contraceptive care may aid individuals in accessing contraception generally, but our findings indicate that these sites may fall short in linking those users to a specific desired method that may be expensive. These findings may also indicate that those who did not receive any recent SRH care had the least interest in using a different method (among users) or any method at all (among nonusers). Our findings may also indicate that cost is not the driving factor for nonuse of contraception. At the same time, increased attention and support for helping contraceptive users to navigate remaining cost barriers to realize their preferences is paramount, especially for low-income individuals.

In addition to individuals’ access to contraceptive care, their experience at the contraceptive care visit can influence fulfillment of their cost-related contraceptive preferences. For contraceptive users, those who had received person-centered contraceptive counseling had lower levels of unfulfilled contraceptive preferences due to cost, highlighting the key role that the patient-provider relationship plays in individuals being able to realize reproductive autonomy in method choice, regardless of their ability to pay. These national-level findings support smaller studies [18] and highlight the importance of tracking patient-centeredness in contraceptive care as one aspect of quality family planning care delivery, a measure of which was recently endorsed by the National Quality Forum (NQF) [19]. In contrast, nonusers of contraception who had received recent contraceptive care demonstrated no such relationship, which may indicate that person-centered contraceptive counseling is important but not sufficient as a buffer to cost barriers for nonusers who want to become users.

Beyond contraceptive access and care experiences, some individual-level characteristics played a role in reports of unfulfilled contraceptive preferences. Compared to those using LARC methods, users of all other contraceptive methods have higher rates of unfulfilled contraceptive preferences due to cost. Many LARC users are satisfied with their IUD or implant [20], which are also some of the most expensive contraceptive options available. Although these data do not shed light on which methods individuals would prefer to be using, lowering the cost of LARC methods would be one avenue through which non-LARC users could realize LARC method preferences. Those using non-LARC methods may be doing so partially due to their less expensive nature, but also for a variety of other reasons, including preferring user-controlled methods rather than those that require seeing a provider to start and stop, wanting shorter-term methods, and liking nonhormonal options among others [21], [22], [23]. Still other non-LARC contraceptive users may prefer to use other expensive methods—such as permanent ones—but these expensive options may also be out of reach.

Among contraceptive users, those who identified as foreign-born had higher levels of unfulfilled contraceptive methods due to cost, even when accounting for subsidized access to and experiences of contraceptive care. Foreign-born women also have lower levels of health insurance coverage, lower levels of receiving any SRH care, and higher levels of paying out of pocket for that care than United States-born women [24], highlighting that these access metrics may be playing a role in impeding foreign-born women's ability to realize their contraceptive preferences without cost considerations. Efforts to support all individuals in choosing and using their preferred method regardless of cost through inclusive and equitable approaches are warranted to address these gaps.

Our analysis has several key limitations to note. These data likely do not fully represent the breadth of contraceptive strategies or multiple methods employed by users [25], as analytic variables focused on singular method use. Our analytic outcome is specific to the context of cost considerations; determining how these preferences intersect with other considerations related to contraceptive choice is paramount. In addition, the NSFG does not have information regarding which methods contraceptive users would prefer to be using, so our interpretations of this outcome are limited. Finally, given the cross-sectional nature of our analysis, findings do not necessarily imply a causal relationship.

Minimizing the gap between individuals' preferred and actual contraceptive use is one key aspect of helping individuals achieve reproductive autonomy as it relates to contraceptive choice. Factors related to access to contraception at the systems level—specifically health insurance coverage, whether and where SRH care is obtained, and experience of contraceptive care—impact whether individuals can overcome cost barriers to realize their contraceptive preferences. Our study highlights that both health insurance coverage and patient-centered contraceptive care help contraceptive users to overcome cost barriers to their cost-related contraceptive preferences. Nonusers of contraception face broader hurdles to overcoming cost-related barriers to contraceptive preferences. More research is needed to understand broad contraceptive preferences that go beyond cost considerations and which take into account other factors related to access that may constrain or support people in realizing contraceptive preferences, such as the legacy of racism and xenophobia and discrimination in health care settings [26,27]. Finally, given the COVID-19 pandemic and the resulting impacts on both the delivery of contraceptive care [28], [29], [30] and in delays in access to this care [31], our study highlights the importance of continuing to support evolutions in the health care system that ensure a broad range of contraceptive options are available to enable individuals to realize their contraceptive preferences regardless of site of care or method cost.

## Footnotes

1 All NSFG respondents self-report their current gender at the time of interview, which determines the questionnaire they are routed into. For this analysis, we use the terms “women” and “female” to mirror NSFG language, but not all respondents included in the female NSFG respondent file may identify in this way.

2 https://www.cdc.gov/nchs/data/nsfg/NSFG_2013-2015_Summary_Design_Data_Collection.pdf

3 http://www.cdc.gov/nchs/nsfg.htm

4 SRH care broadly includes gynecologic care, pregnancy care, STI care, and contraceptive care. Contraceptive-specific SRH care includes receiving a method of contraception, contraceptive counseling, or a check-up related to contraceptive use (including sterilization operations and emergency contraceptive use).

5 Respondents who indicated having received contraceptive care in the past year at an “other” type of site (including employer or company clinic, hospital inpatient care, emergency room, urgent care center, and some other place) were removed from this analysis due to the small N and relative heterogeneity among these types of care (*N* = 202/8206 in 2015–2019).
